# Ten quick tips to build a Model Life Cycle

**DOI:** 10.1371/journal.pcbi.1012731

**Published:** 2025-02-03

**Authors:** Timothée Poisot, Daniel J. Becker, Cole B. Brookson, Ellie Graeden, Sadie J. Ryan, Gemma Turon, Colin Carlson

**Affiliations:** 1 Département de Sciences Biologiques, Université de Montréal, Montréal, Québec, Canada; 2 Québec Centre for Biodiversity Science, Montréal, Québec, Canada; 3 School of Biological Sciences, University of Oklahoma, Norman, Oklahoma, United States of America; 4 Yale University School of Public Health, New Haven, CT, United States of America; 5 Massive Data Institute, Georgetown University, Washington, DC, United States of America; 6 Department of Geography and Emerging Pathogens Institute, University of Florida, Gainesville, Florida, United States of America; 7 College of Life Sciences, University of KwaZulu Natal, Durban, South Africa; 8 Ersilia Open Source Initiative, Barcelona, Spain; SIB Swiss Institute of Bioinformatics, SWITZERLAND

## Introduction

Managing the development of a model through its lifecycle is as key to reproducible research as data management planning. Following-up on recent articles outlining the foundations of model development in computational biology [1,2], our aim is to provide guidance about the management of models in a way that is inspired by best practices in data management. Using a robust data management plan is a cornerstone of modern data stewardship [3]. Thinking of research data as living objects that are inextricably tied to the researchers that collect them, can grow over time, and be re-used by others has the dual advantage of establishing a higher standard of care for data and facilitating their use and adoption by the community [4,5]. Surprisingly, we have not always applied the same analysis to the models into which we feed these data. Although there is a wealth of literature suggesting best practices for the use and development of predictive models, they focus on checking the model correctness [6], establishing the correct mathematical approaches [1], adopting good simulation workflows [2], properly storing and manipulating data [7,8], or ensuring that our work with data, and anything downstream of this work, is ethical [9].

**Fig 1:**
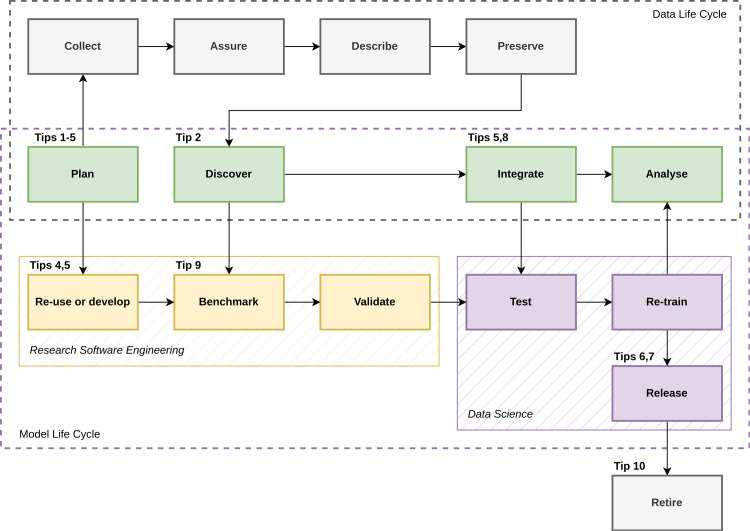
The Model Life Cycle. The Data Life Cycle (the “Analyse” to “Plan” feedback has been omitted for clarity) is split into two parts, with data collection-specific tasks (top row, grey) and shared data collection/analysis parts (middle row, green); the Model Life Cycle (bottom box) is integrated into the Data Life Cycle, with model development-specific tasks (left, yellow), and model application- and model interpretation-specific tasks (right, purple). This division of steps also outlines broad divisions of effort in the team (grey: experimental work; yellow: research software engineering; purple: data science and machine learning operations; green, collective effort).

All of these considerations are extremely important! However, a gap remains in the biological sciences literature that guides people towards good practices in modelling: just like data, models have their own life cycle. By recognising how one’s model fits within the life cycle of the data (or at least, ensuring that the Model Life Cycle is understood), we can identify opportunities to foster new collaborations, encourage better practices in data analysis [10], and ultimately accelerate research. In this manuscript, we introduce the Model Life Cycle for biological data-driven research (Fig 1) and develop a series of 10 quick tips aimed at facilitating collaborations between data collectors, curators, users, and modellers, as well as maximising the potential for re-use of models. We explore the idea of a Model Life Cycle, starting from the assumption that it will address machine learning (ML) models, i.e., models that can be trained and deployed iteratively, and whose focus is on prediction of quantifiable phenomena. Specifically, we are interested in clarifying the use of models in large, interdisciplinary groups, where the actual modelling exercise may involve only a subset of the group (e.g., with others collecting and standardising data). Nevertheless, we have written the recommendations to broadly apply to varied practices of modelling in the life sciences.

Data are most often collected by those who need to use the data; these end users, as defined by the data collectors, define the requirements for what metadata are collected and the research methods applied to data collection. Therefore, the data collected are inherently tied to the use case. By contrast, the developers of models are very commonly not the users of those models, particularly not as model development often requires many developers, including machine learning operations, infrastructure engineering, data engineering, parameterisation and testing, and user interface development focussed on surfacing models for end users. This disconnect can mask the influence of decisions made deep in the stack by developers who have not been communicated a full picture of the downstream end users or use cases for the model. For example, decisions made about the use of specific types of differential privacy or other privacy enhancing technologies in a model used to evaluate survey data may prevent the use of the model for time-series analyses. This is particularly true when changes over time for specific individuals are required to assess the impact of interventions. Yet, differential privacy may require to decouple the parameters from the specific populations to which the interventions were tied.

Data are not (always) goods that arrive at a modeller’s doorstep. That is, the work of the modeller should not be entirely decoupled from the process by which those data are collected. In this manuscript, by building on the existing formalism of a life cycle for scientific research data, we outline a way to integrate the model development as a core component of the research process. The purpose of this life cycle is to divide the labour of model production and deployment among different groups, and offer concrete recommendations for best practices in ensuring that data collection and model development proceed together.

## Tip 1: Remember that models are stepping stones

Models are a step between the research question and solution [11], but we need to establish that modelling involves different skills from the research itself. In the field of biodiversity conservation, e.g., models involving ML can intervene to mediate typically disconnected remote sensing and participatory approaches [12]. Of course, not all contributors to the research process will interact directly with models, which is particularly true when models become more complex (which is to say, when training and deploying these models requires specific technical skills that are not those involved in the research process itself). For this reason, it is expected that the process of establishing a good model will have to branch from the data life cycle, to include contributors with expertise in domains that are tied to the conception, production, and operationalisation of predictive models. In Fig 1, we have outlined a potential branching and merging process for the Model Life Cycle. This schematic is meant to be a guiding principle that must be adapted to each specific research context.

## Tip 2: Re-use (other’s) data before you use (your own) data

Applying the tips in this manuscript should lead research groups to a robust modelling strategy all while the data are being generated. However, there are ways to kick-start the learning process even in the absence of the actual data to which the final model will be applied. Broadly speaking, this can take the shape of transfer learning [13], i.e., the training of a model on an initial situation, to minimise the cost it would take to re-train it on a new (but similar) problem. This approach hinges on the fact that some systems are inherently close to one another [14], and can therefore be well approximated by the same initial model. This does not remove the need for specifically re-training the model to the actual dataset, but it can help establish a reasonable working model early in the process.

In some situations where the generated data will follow the same structure as already available data, these existing datasets can be used to establish benchmarks, e.g., before applying a model predicting trophic interactions between mammals of North America, Strydom and colleagues [15] confirmed the lack of over-fitting and the performance of this model using similar data from Europe. Although this approach is reliant on the availability of data with the same structure (and ideally a similar collection process or underlying assumptions, which cannot be determined by the modellers alone and must involve data producers), when possible, it allows establishing most of the predictive pipeline before data collection starts.

## Tip 3: Design models before using models

All models require data. Defining the relationship between the data you are using, and the model, is a critical first step when establishing the role of modelling in your research design. Is the goal of your modelling to capture the variance of the data, to test a modelled process using new data, to validate a model using a new dataset, or training a model on a subset of the data and validating the model with the remaining data? Once you can determine the role of the data in your modelling adventure, then you can begin to assess what kinds of modelling methods and model performance measures will be meaningful. It should be the exception, rather than the rule, that a problem requires the creation of an entirely new model to be solved. Defining the research question at hand, and describing the processes involved and what outcomes (i.e., the data) are needed, is Step 0 in the formulation of any model [16].

In most cases, the actual process of refining a model implies identifying an algorithm based on the type of problem (e.g., classification, regression, unsupervised learning) and then outlining a strategy to oversee the training and validation of this model, including using these outcomes to define the data sources for the modelling. Remarkably, much of this work can be done without even having seen the data on which the model will be applied. For example, the *MLJ* library in Julia [17] enables the user to establish the specification of the features and labels, and returns a list of algorithms that support this combination of types. By identifying the data types and data sources needed, as well as preparing metadata required for further analysis, colleagues in charge of the modelling step can start making substantial progress during data collection. Ideally, most of the boilerplate code can be written (or adapted from prior projects), and validation/visualisation solutions agreed upon, well in advance of the application of the model to the data. For more advanced cases, synthetic datasets [18], where realistic-looking datasets are reconstructed from published sources or simulated from similar data [19], can be used. Importantly, building the model in advance protects against the temptation to adapt the model to the desired results: by reasoning about the best way to handle (future) data, teams can avoid decisions that are biased by pre-existing knowledge of the results when elaborating the models alongside the data analysis.

## Tip 4: Re-using models is fine

In addition to the availability of data, the repertoire of already published models to solve a specific family of biological questions can be leveraged to develop novel predictive pipelines and insights. For example, Becker and colleagues [20] re-used multiple models from community ecology to predict potential bat hosts of beta coronaviruses, at a time when observational and experimental validation of some of these host species was ongoing. By using not only the existing code for these models, but also the previous discussion of their caveats and advantages, the research effort shifted from model production to model integration and analysis, accelerating the entire process considerably.

Most predictive tasks do not require much in terms of methodological development, and by drawing on previous efforts for related problems, research groups can more tightly integrate their results with the existing literature. This facilitates the assessment of the relevance and validity of the approach and, when (with rule 9) it identifies inadequacies in the previous models, provides a strong statement of need for future methodological work. For models where initial conditions or specific hyper-parameters are key, using formats that track ML experiments (like e.g., *Tensorboard*) is important.

## Tip 5: Consider data architecture and access

Ask yourself: what will *all* the data the model will be applied on look like? If they are measurements, what was the measurement process, and how will your model account (or not) for observation processes and errors? If they exist as flat (i.e., static) files, or will be pulled from (possibly relational) databases, what properties will be important to your modelling adventure? Information about data storage will be a necessary plan of the Data Life Cycle, in ways that will span the entire research group, starting with the management of experimental and observational data [21]. The shape of the data will not only determine what models are appropriate, but also help the researchers anticipate the runtime requirements of the model; file-system based versus relational database versus graph database storage can lead to profound differences in the system requirements to run a model. Data transformation and re-shaping steps can be extremely taxing, notably when they incur many input/output (writing to and reading from disk) operations. By engaging in a discussion about the data representation requirements, modellers ensure they design models that will be able to accept the empirical data, while data producers ensure that they can provide data in a way that minimises the computational costs.

Such conversations can also assist with reconciling different datasets into a common model, like matching different host–pathogen association data to a common host and pathogen taxonomic backbone [22]. Clear group-wide agreement about the architecture of data also helps when the data are expected to be regularly updated [23]; if the data collection is part of an ongoing process (either through sampling or through the contribution to community data sharing platforms), clear expectations about data structure and handling will ensure the long-term viability of the models and their application.

Finally, conducting a painstaking inventory of the data provenance will also help establish intellectual property and/or research credit, as is appropriate for the data in question. Although intellectual property is important for potential commercial applications, it is also morally indispensable in many applied scientific cases, such as when considerations around the data involve Indigenous data sovereignty [24,25] or when the privacy of data collectors can be compromised [26,27].

## Tip 6: Sharing the code is good

Verbal descriptions of the model often fail to communicate the full nuance of an analysis. As models are primarily computational artefacts, sharing the code through which the model is trained and its predictions made boosts the potential for not only auditing, but also re-use. In ecology and evolution, code sharing (across all practices of research that generate code) is associated with higher citations [28], an effect that persists even when controlling for the journal in which the articles are published. Empowering the community to re-use one’s work is a way to build a scientific reputation. Low sharing of code is also preventing scientific progress: it is the main obstacle to the reproducibility of computational studies [29]. Importantly, adding an Open Source license will allow future modellers to re-use one’s work appropriately [30].

There are still strong barriers to code sharing [31]. Nevertheless, they should be less severe for most ML-based models: this code is typically written by relying on high-level wrappers around ML packages (*MLJ*, *Keras Core*, *PyTorch*, etc.), which involves chaining together functions rather than the development of genuinely new functionalities. We should expect to see the practice of code sharing increase in the near future. Indeed, the FAIR principles of data sharing and re-use [32] have recently been adapted to the specific challenges of research software [33].

## Tip 7: Sharing more than the code is better

Code sharing enables the re-use of models, and we expect this will increase through journal mandates [34] and funding agency recommendations [35], thereby facilitating the application of tips 2, 4, and 6. However, models are more than their code. Parameterised (trained) models can be serialised to an object that can have a well-documented data format, such as *tflite* or binary JSON [36]. These models can then be loaded in a language-agnostic way, thereby providing access to the *actual* model, as opposed to the *potential* model (represented by the code to specify and train it). Ultimately, this approach enables researchers using a different ML software stack to re-use already trained models. In practice, the sharing of trained models is already happening for deep learning-based approaches, like e.g., BirdNet [37] or re-trained ResNet50 for fauna detection [38].

For models that are likely to have far-reaching usability, advanced model sharing platforms like Hugging Face are becoming the *de facto* standard in Natural Language Processing [39]. The practice of model sharing on these platforms is now mature enough that there are published recommendations [40]. An interesting recent example is the release of BioCLIP [41], a computer vision model that matches images to taxonomic names, with additional constraints on species pool, taxonomic rank, etc. A model of this scope is likely useful to all biodiversity scientists relying on automated image analysis, but it requires resources for training that would make its adoption difficult otherwise.

In addition, complex models with multiple data streams rely on equally complex software environments that are best reproduced via containers, to avoid software version and/or operating system incompatibilities. Others have written extensively about the necessity of containerisation for the reproducibility of these software environments [42], but learning how to fully containerise models takes time and effort, which is drastically underappreciated and undervalued in the publication-based reward systems of research. Despite these challenges, without these key tools, many analysis pipelines become essentially unusable to others. Docker stacks (and other container-based software) are near-ubiquitous in commercial ML pipelines, and have proved essential for forecasting tasks and competitions [43,44] as well as real-life forecasts that inform management decisions [45]. Containerising parts of or all of one’s forecast will inevitably make it much easier for others to (a) examine the work effectively and (b) implement valuable re-use strategies such as in tips 2 and 6.

## Tip 8: Consider data ontologies

Some communities of practice may have developed specific data or metadata representations. In ecology, e.g., the Darwin core [46] and the Humboldt core [47] provide standardised data representations for taxonomic and occurrence data, respectively. More generic metadata may also be released in the Ecological Metadata Language [48], which provides a nomenclature for the description of ecological studies. Recently, the Ecological Forecasting Initiative introduced a new superset of the Ecological Metadata Language to describe iterative forecasts [49]. These attempts at standardising the communication of data formats and vocabularies are useful, as they remove ambiguities around the content of the dataset, and therefore facilitate cross-team and cross-field collaborations. Recent research emphasises that adhering to ontologies (controlled vocabularies with community-defined terms, which go beyond free-form description of metadata) can make textual information easier to parse, which will enable better data extraction and reuse by systematic reviews or text mining projects, or even potentially the productive use of Large Language Models trained on domain-specific tasks [50].

In some cases, and particularly, when working on large and/or interdisciplinary modelling projects, it cannot be assumed that researchers will organise their data around a shared ontology or taxonomy. For example, when referring to geography, a researcher studying pathogen spillover from wildlife may rely heavily on polygon representations of species distributions. If assessing risk from this spillover event on relevant human populations, these populations will be defined by geopolitical boundaries. Identifying a shared or minimum standard shared unit (e.g., latitude and longitude) can be effective when moving between these datasets as an alternative to assigning or mandating a shared ontology. In some cases, knowledge graphs or other methods of integration based on semantic rules can be useful.

## Tip 9: Decide on acceptable performance before you start

Once you have determined the goal(s) of the model, check that you decide on the acceptable practices for assessing performance to align with the goal(s). In some specific modelling contexts, we can define a priori acceptable performance. Take the example of a model predicting the presence, or absence, of a species in a location. Depending on how this information will be used, classifiers with the same overall measure of performance may not be as informative to their end users (this, notably, calls for a careful and exhaustive description of the validation and testing strategy, and a plain language summary of how and why performance was assessed). For an invasive species, where the environmental cost of a false omission is high, prioritising models with good negative predictive values will make more sense. In contrast, for a threatened species, where preserving a patch of unsuitable habitat leads to inefficient allocation of resources and effort, it would make sense to instead prioritise a classifier with a good positive predictive value. Finally, to think about the distribution of a species in a way that is more detached from specific interventions (e.g., for macroecological research), reaching a balance between these two types of error may be the most desirable outcome.

Picking the model that is the fittest for downstream, targeted purpose is a decision that must account for both the model and the purpose. By engaging in a reflection about what makes a model useful for a specific task, which can be done before talking about the specifics of the model, research groups will ensure they will be able to decide on the suitability of the model when it is finally trained. In addition, some fields may have their own state-of-the-art benchmarks, e.g., the Therapeutics Data Commons initiative [51,52] publishes a benchmark that will let modellers know whether their current best effort qualifies as “good enough”.

## Tip 10: Retire your models

Models are built to answer a specific question, which is framed by a rich context: data availability; data quality; expected type of answer; spatial, phylogenetic, or temporal resolution; and domain knowledge about the phenomenon to be modelled. As these elements change, we expect that models will lose relevance, which introduces the question of when models should be maintained and when they should be retired. Changes in the quantity of data can often be solved with re-training, e.g., if a model recommends potential hosts of a family of viruses, the model can incorporate de novo sampling, which serves both as post hoc validation and as an augmented training set [20]. However, changes in the type of data (e.g., quantifying tree growth from visual inventories and then from remotely sensed data) may require an entirely new type of model. The emergence of new modelling paradigms can also (over a longer time-course) replace previous generations of models, e.g., the recent *GraphCast* weather forecast model [53], through the use of innovative deep learning techniques, outperforms current state-of-the-art weather forecasting models.

Models are fundamentally encapsulating our best attempt at representing reality. Our understanding of the structure that a model purports to describe evolves with time (e.g., we can refine mechanisms of pathogen transmission cycles to include more components as we learn how to measure them [54], or the parameterisation of components takes different shapes (e.g., transitioning from linear descriptions of systems to non-linear). Building on models allows them to evolve, perhaps even displacing ‘older’ formulations in favour of improved descriptions of mechanistic processes. In this scenario, the model life span has a natural arc. Sometimes models such as this can be maintained as baseline models to demonstrate improvements (of fit, of form, of internal or external validation) as models evolve.

## Conclusion

Tackling the most pressing scientific challenges requires the best data and the best models, and we are far past the point where it is reasonable to assume that a single researcher (or indeed a single team) will be able to deliver on both. The optimal way forward is to develop templates for healthy, productive collaborations between data- and model-centric workflows. Because the Data Life Cycle has a proven track record of systematizing the way we think about the changing shape of data throughout a project, here we propose that we can overlay a Model Life Cycle on top of it. We hope that the overlaying of these two cycles can generate higher impact, more reproducible, and strifeless research collaborations. The illustration of the Model Life Cycle we present in Fig 1 is a template that must be tweaked to respect the specific considerations and contingencies of various research groups; nevertheless, it indicates how we can be a little more systematic in our approach to bridging data and models.
